# Quantification of chemical and mechanical bioerosion rates of six Caribbean excavating sponge species found on the coral reefs of Curaçao

**DOI:** 10.1371/journal.pone.0197824

**Published:** 2018-05-30

**Authors:** Didier M. de Bakker, Alice E. Webb, Lisanne A. van den Bogaart, Steven M. A. C. van Heuven, Erik H. Meesters, Fleur C. van Duyl

**Affiliations:** 1 Wageningen Marine Research, Den Helder, Netherlands; 2 Marine Microbiology and Biogeochemistry, NIOZ Royal Netherlands Institute for Sea Research and Utrecht University, Texel, Netherlands; 3 Ocean Systems, NIOZ Royal Netherlands Institute for Sea Research and Utrecht University, Texel, Netherlands; 4 Marine Biology Research Group, Ghent University, Ghent, Belgium; University of Genova, ITALY

## Abstract

Excavating sponges are among the most important macro-eroders of carbonate substrates in marine systems. Their capacity to remove substantial amounts of limestone makes these animals significant players that can unbalance the reef carbonate budget of tropical coral reefs. Nevertheless, excavating sponges are currently rarely incorporated in standardized surveys and experimental work is often restricted to a few species. Here were provide chemical and mechanical bioerosion rates for the six excavating sponge species most commonly found on the shallow reef of Curaçao (southern Caribbean): *Cliona caribbaea*, *C*. *aprica*, *C*. *delitrix*, *C*. *amplicavata*, *Siphonodictyon brevitubulatum* and *Suberea flavolivescens*. Chemical, mechanical and total bioerosion rates were estimated based on various experimental approaches applied to sponge infested limestone cores. Conventional standing incubation techniques were shown to strongly influence the chemical dissolution signal. Final rates, based on the change in alkalinity of the incubation water, declined significantly as a function of incubation time. This effect was mitigated by the use of a flow-through incubation system. Additionally, we found that mechanically removed carbonate fragments collected in the flow-through chamber (1 h) as well as a long-term collection method (1 wk) generally yielded comparable estimates for the capacity of these sponges to mechanically remove substratum. Observed interspecific variation could evidently be linked to the adopted boring strategy (i.e. gallery-forming, cavity-forming or network-working) and presence or absence of symbiotic zooxanthellae. Notably, a clear diurnal pattern was found only in species that harbour a dense photosymbiotic community. In these species chemical erosion was substantially higher during the day. Overall, the sum of individually acquired chemical and mechanical erosion using flow-through incubations was comparable to rates obtained gravimetrically. Such consistency is a first in this field of research. These findings support the much needed confirmation that, depending on the scientific demand, the different approaches presented here can be implemented concurrently as standardized methods.

## Introduction

The existence of tropical coral reefs relies on an ongoing biogenic precipitation of calcium carbonate (CaCO_3_) by calcifying organisms (most importantly scleractinian corals) that is exceeding erosional forces [1–7]. Live corals, however, have been diminishing worldwide at unprecedented rates following phenomena such as reduced water quality, climate change and declined herbivorous pressure (e.g. [8, 9–14]). Reefs in the Caribbean and Gulf of Mexico, in particular, have been heavily impacted in the past decades. Here coral cover was reduced, on average, to a mere 16% [15–17]. Alarmingly, the observed decline can largely be accredited to the mortality of formerly dominant framework-building species (i.e. *Acropora* spp. and *Orbicella* spp.) which are especially affected by direct or indirect anthropogenic stressors [18–20]. Whilst the main reef-builders appear largely unable to cope with the current level of global and local disturbance [21], the changing reef environment seems to favour, among others, bioeroding organisms) (For an overview see [22]: Table 3, Supplementary data S4).

Within the bioeroding community excavating sponges are frequently the dominant macroboring organisms [23–25]. On Caribbean reefs sponges can be responsible for as much as 90% of the total macrobioerosion [26]. Murphy et al. (2016) [27] describe two main strategies by which these sponges erode their substratum: (1) gallery-forming, where the sponge progressively works its way down, and (2) cavity-forming, where the sponge penetrates the limestone and forms chambers inside the substrate with often only the inhalant and exhalant fistules being visible on the surface. Many gallery-forming sponges harbour high densities of dinoflagellate zooxanthellae (i.e. *Symbiodinium* spp.) and as such their boring strategy allows for optimal light exposure [28]. Excavating sponges erode hard substratum through a combination of chemical dissolution and mechanical removal of CaCO_3_ fragments (chips, distinctly recognizable by their scalloped surface) [29–31]. Previous efforts studying the contribution of the individual components suggest that the mechanical fraction generally accounts for the majority (up to 98%) of total erosion (e.g. [29, 32, 33–35]), although an opposite pattern has been described by Zundelevich et al. (2007) [36] for *Pione vastifica* (β-stage). The product of mechanical erosion by sponges contributes significantly to the total pool of fine silt-sized sediment on coral reefs ([37], and references therein), which is essential to reef cementation and stabilization [38].

There is ample scientific support that excavating sponges respond positively in both abundance and boring activity to factors such as deteriorating water quality (e.g. eutrophication), ocean acidification, elevated sea surface temperature and increased availability of substratum following coral mortality ([22] and references therein). Nevertheless, they are rarely incorporated in standardized reef surveys and experimental work is restricted to a limited number of species. This is in part due the endolithic life-style of the sponge. The few surveys that do include excavating sponges, such as the *ReefBudget* method [39], have to rely on scarce and often inconsistent data with respect to rates of bioerosion. Currently, techniques to estimate these rates are inconsistent and the procedures involved are tedious and prone to methodological bias.

The available published data on total estimated sponge bioerosion displays tremendous variation with rates ranging from 0.3 kg m^-2^ y^-1^ to 29.5 kg m^-2^ y^-1^ ([40]: S3 Table]). Density of the invaded substratum has a considerable impact on the attained rates (e.g. [41, 42]), but also factors such as studied species, boring strategy, developmental stage (α, β, γ or δ-stage) and applied methodology can cause significant variability ([40] S3 Table].) In early work erosion rates were generally determined based on weight loss of the host substratum over a designated period [42–45], occasionally in combination with chip collection experiments to quantify mechanical erosion. Buoyant weight has been commonly implemented to estimate coral growth rates, but has also proven a good proxy to quantify rates of sponge bioerosion (e.g. [42, 46]). A major advantage of the buoyant weight technique is that the studied material can remain submerged during the weighing process. This approach on itself, however, is not selective to means of erosion (i.e. chemical or mechanical), which is relevant, for instance, when studying the effect of ocean acidification on the chemical fraction of sponge bioerosion. Recent efforts have therefore implemented incubation techniques to study both components separately. By incubating sponge infested substratum it is possible to determine chemical bioerosion based on the increase in alkalinity of the incubation-water over time [36]. This technique has now been widely adopted to quantify effects of environmental change and future climate scenarios on rates of bioerosion (e.g. [34, 35, 47, 48, 49]). A major disadvantage of this method, however, is that sponges are kept in a limited body of water for a relatively long time, which could strongly affect the metabolism of the sponge following food and oxygen depletion and the accumulation of waste products [50]. Moreover, the lack of understanding of the processes underlying mechanical chip removal adds great uncertainty to previously quantified mechanical bioerosion rates. Indeed, periodic expelling of chips and/or contamination of filters by other material may induce error in the quantification of mechanical bioerosion (e.g. [34, 35]). Evidently, the considerable variation in published sponge bioerosion rates stresses the need for consistency in applied procedures.

In the present study, the chemical and mechanical bioerosion rate of six boring sponge species that dominate bioeroding community on the southern Caribbean reefs of Curaçao was determined. Chemical rates were estimated using flow-through incubation techniques, which were compared to previously used standing incubations. For mechanical rates a distinction was made between actual chips and other CaCO_3_ material removed by the eroding activities of the sponge over a designated time span. These approaches were then compared to conventional buoyant weighing procedures. We aim to optimize techniques to quantify bioerosion and to determine variation in chemical and mechanical bioerosion of sponge species with different functional traits (e.g. presence/absence of photosymbionts, boring strategies and developmental stages) and in light of a changing reef environment.

## Materials and methods

### Ethics statement

All research was conducted under research permit (#2012/48584) issued by the Curaçaoan Ministry of Health, Environment and Nature (GMN) to the CARMABI Foundation.

### Study area and sample collection

Sponges were collected on reefs at the leeward side of the island of Curaçao (southern Caribbean) in February 2017. Reefs here are characterized by a reef flat and a drop-off that gradually slopes down from approximately 10 m depth [51]. Samples were collected at Snake Bay (12°8’N, 68°59’W), Piscadera Bay (12°7’N, 68°58’ W) and Directors Bay (12°3’N, 68°51’W) between 5 and 15 m depth. Sponge infested cores of the most dominant species were removed from old dead coral substratum using a pneumatic drill with hole-saw (inner diameter 45 mm). Collected cores were cleaned and non-sponge organisms removed. The collected material was kept in large flow-through aquaria in the research facilities of the Carmabi Research Station. Cores were left to acclimatize and recover for at least seven days. Full tissue regeneration was observed within this period (see also [52]).

### Species description

**Fig 1:**
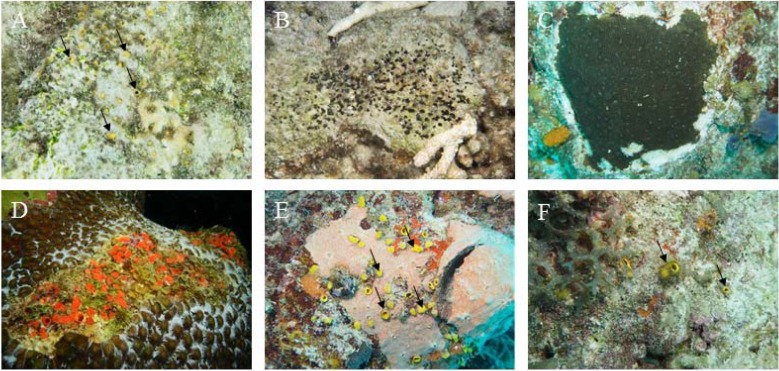
In situ close-up of the six studied excavating sponge species. **A.** *Cliona amplicavata* (α-stage) **B.** *Cliona aprica* (α-stage) **C.** *Cliona caribbaea* (β-stage) **D.** *Cliona delitrix* (α-stage) **E.** *Siphonodictyon brevitubulatum* (α-stage), please note that the yellow fistules of *S*. *brevitubulatum* are surrounded by a different sponge species (pink coloured) **F.** *Suberea flavolivescens* (α-stage). Black arrows in **A**, **E** and **F** point out oscula and ostia of the studied sponges.

A total of six excavating sponge species were collected including two gallery-forming species (Fig 1): *Cliona caribbaea* (Carter 1882) and *C*. *aprica* [28], three cavity-forming species: *C*. *delitrix* [28], *C*. *amplicavata* [53] and *Siphonodictyon brevitubulatum* [28] and *Suberea flavolivescens*, a species that formed a dense network through the carbonate substratum (from here onwards referred to as network-forming). This is, to our knowledge, the first scientific description of *C*. *amplicavata* and *S*. *flavolivescens* on the reefs of Curaçao. Both gallery-forming species belong to the *Cliona viridis* species complex [54, 55] and harbour high densities of dinoflagellate zooxanthellae [28]. *C*. *delitrix* has also been reported to hold zooxanthellae, but in considerably lower densities [56]. The other three species do not appear to harbour symbiotic zooxanthellae [28, 53]. α-Stage specimens were collected for *C*. *aprica*, *C*. *delitrix*, *C*. *amplicavata*, and *S*. *brevitubulatum* and β-stage specimens for *C*. *caribbaea* and *S*. *flavolivescens*. The α-stage of *C*. *delitrix* (often confused with *C*. *laticavicola*) [57] was chosen since the β-stage was only recently observed for the first time on Curaçaoan reefs [58]. To allow for inter-specific comparison, all specimens, with the exception of *C*. *amplicavata*, were collected from infested dead *Orbicella* spp. coral, which is the most common substratum on many Caribbean reefs [59, 60] and of average skeletal density (~1.8 g cm^-3^) [61]. *C*. *amplicavata* was only found in *Acropora* spp. rubble. Same sized uncolonized cores were collected from old *Orbicella* spp. and *Acropora* spp. skeleton to serve as control substratum throughout all experiments allowing adjustment for possible dissolution through micro-bioerosion, abrasion caused by handling and accretion due to the potential presence of calcifying organisms (e.g. [35]). In order to prevent decomposition of *S*. *brevitubulatum*, the majority of the soft internal tissue was removed and cores were left to recover for several additional days before being used experimentally. Species identification was confirmed by spicule morphology analysis.

### Experimental set-ups

Sponge bioerosion was measured in different experimental settings in large flow-through (~5 L min^-1^) holding tanks. We used 500 mL incubation chambers consisting of a transparent body with detachable top and bottom. The top contained an electrically driven magnetic stirrer to ensure thorough homogenisation of the incubation water. The incubation chambers were used in a standing mode (ST) without water flowing through and in a continuous flow-through mode (FT). In addition and for comparison total bioerosion was also determined gravimetrically by applying the buoyant weight (BW) technique [62, 63] to hanging cores. Debris from these cores was collected to determine the mechanical bioeroding capacity of the sponges.

With the conventional **standing incubation** the effect of incubation time on rates of chemical erosion was tested for two sponge species. *C*. *caribbaea* (symbiont-rich) and *C*. *delitrix* (symbiont-poor) infested cores were incubated for 1, 2, 4 and 6 h in fully enclosed incubation chambers. Two cores were placed in each chamber which was sealed air-tight to avoid gaseous exchange with the external environment. Chambers were largely submerged to prevent temperature anomalies. Temperature (°C) and oxygen (%) were monitored continuously over the course of the incubation using a PreSens O_2_ sensor (Fibox 4, PSt3). Incubations at night were carried out for a maximum of 3 hours to avoid oxygen levels declining too much (> 80% loss of O_2_ saturated water). Samples for alkalinity (~250 mL) and nutrient (PO_4_^3-^, NH_4_^+^, NO_3_^-^, NO_2_^-^) analyses were collected at the start and the end of each run.

**Fig 2:**
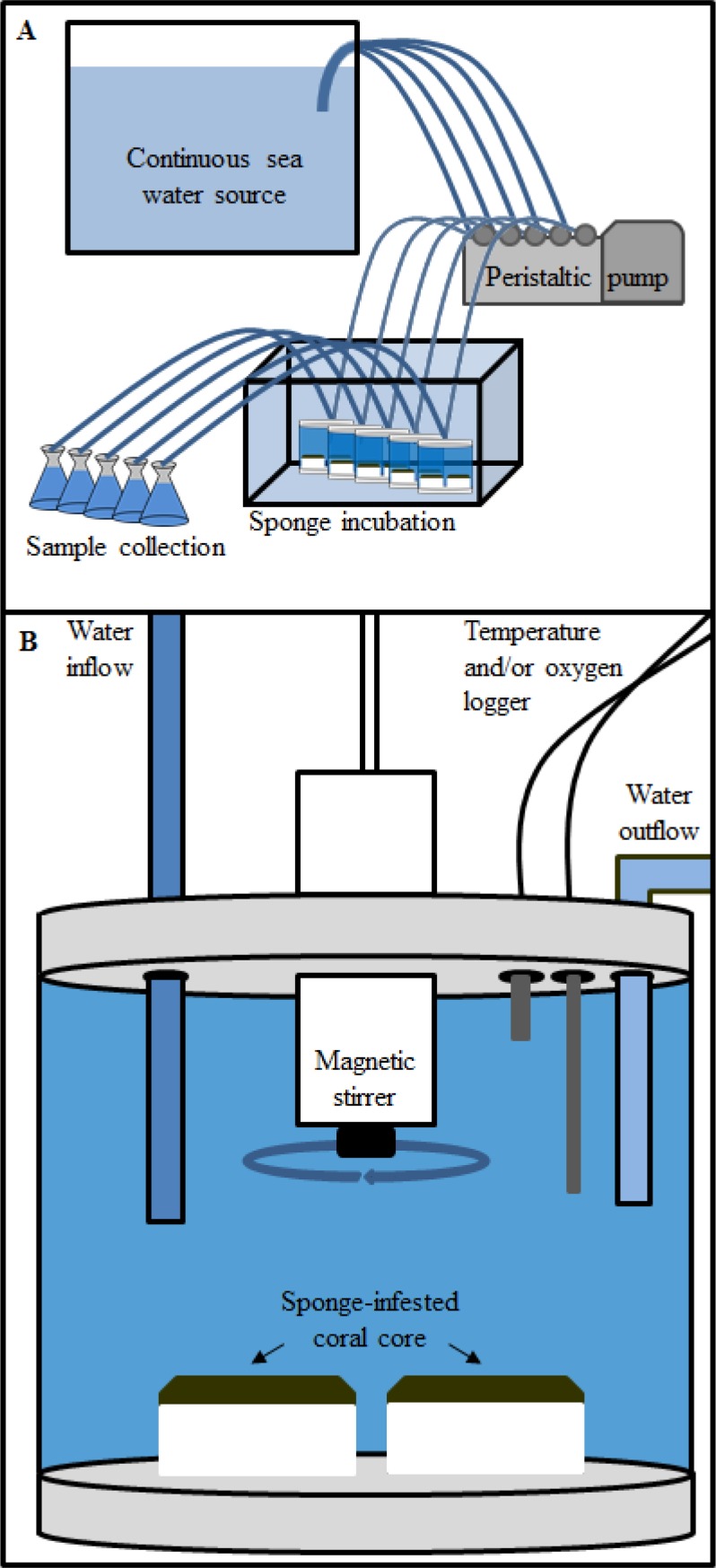
Flow-through set-up. **A.** Overview of the flow-through incubation set-up including the peristaltic pump with adjustable flow-speed continuously pumping fresh sea water through the incubation chambers. The excess water subsequently overflows into collection vessels for nutrient and total alkalinity analyses. Chambers are partly submerged in a holding tank to prevent temperature fluctuation. **B.** Close-up of a flow-through incubation chamber (500 mL) holding two sponge infested limestone cores.

For the **flow-through** set-up the incubation chamber was modified to allow a constant through flow of water (Fig 2). Each chamber contained two sponge-infested or control cores. By means of a peristaltic pump, with adjustable flow-speed, fresh sea water was transported from a reservoir into the chamber where the magnetic stirrer ensured homogenized mixing. The excess water overflowed through flexible Teflon^®^ tubing into a collection vessel. A filter (0.7 μm pore size) was placed on the out-flow tube to prevent loss of particulate material from the chamber. A flow-speed of 1.5 L h^-1^ allowed for continuous refreshment of the incubation water approximately every 20 minutes while still obtaining a reliable signal for water chemistry analyses. This flow-through incubation method ensured a constant supply of food and prevented excessive build-up of waste products, therewith simulating more adequately *in situ* reef conditions. Water samples for alkalinity (A_T_, μmol kg^-1^) and nutrient analyses were collected at the inlet and outlet of the incubation chamber. The system was run for 30 minutes before sample collection started, to allow for an initial full refreshment of the incubation water. This was done to prevent catching a signal that might be affected by the initial incubation water the cores were placed in. Additionally, a possible effect of sponge handling and subsequent acclimatization was herewith largely excluded. Final dissolution rates were calculated from the observed difference in A_T_ which was corrected for the retention time of the water in the incubation chamber. Cores were carefully removed from the FT chamber immediately after water sample collection. Subsequently, all particulate material produced during the entire duration of the incubation (~1 h) was collected as a measure for mechanically removed substratum (mechanical erosion). Chemical and mechanical erosion of sponge infested and control cores was determined at day and night in the FT incubation because erosion rates of sponges, particularly symbiont-bearing species, were found to be affected by light availability (diurnal variation) [34, 64, 65]. Flow through incubations were run for all six sponge species. For two of them, *C*. *caribbaea* and *C*. *delitrix*, results (chemical dissolution rates) of the FT were compared to results of the ST incubation. In order to test the consistency in time of the FT methodology, additional water samples were collected for *C*. *caribbaea* 1, 2, 3 and 4 h after the start of the incubation while the FT system kept running.

Mechanical bioerosion by the six species was also determined over a longer period (7 consecutive days) as part of the **buoyant weight** approach. Three infested cores per species and blank cores were positioned upside down directly above glass funnels (Ø 10 cm) in a large holding tank. Particulate material caught in each funnel (placed in a collection vial) as well as in the FT incubation chamber was collected on pre-combusted (550°C, 3 h), pre-weighed, 0.7 μm Whatman^®^ glass microfiber filters. Filters were subsequently re-combusted (550°C, 3 h) to remove any organic material and re-weighed resulting in an estimate of the particulate material produced over the course of each experimental approach. As an additional analysis, material collected on the filters of the one week experiment was homogenized and subsampled. Scanning Electron Microscopy (SEM) of the subsample was used to estimate the actual contribution of sponge chips, non-chip CaCO_3_ fragments and other material (e.g. spicules, foreign materials). FT filters contained too little material for adequate SEM analysis. Results of both experiments were corrected for the weight of fragments collected from uncolonized control cores to ensure that only the fraction removed by the sponge would be included in estimates of total mechanical erosion.

To determine total bioerosion based on the loss in buoyant mass, the hanging cores were first weighed at the beginning of the 7-day collection period following the BW technique as described by Fang et al. (2013b) [35]. Cores were re-weighed after hanging for three full weeks. All measurements were conducted by means of an electronic weighing apparatus (0.1 mg accuracy). Organic sponge tissue was assumed to equal the density of the ambient sea water so to eliminate the effect of sponge growth. Buoyant weight of the cores was calibrated for fluctuations in seawater density. The change in weight of each core over the three week period was corrected by the change in buoyant mass of the uncolonized control cores and subsequently converted into rates of total sponge bioerosion.

### Chemical erosion

The mass of CaCO_3_ dissolved by each sponge (*ΔM*) was calculated from the change in total alkalinity over the course of the incubation following Eq (1) [32, 36]. Fluctuations in PO_4_^3-^, NH_4_^+^, NO_3_^-^ and NO_2_^-^ can affect ΔA_T_ and are therefore included in the equation as well [47, 66].

$$\Delta M_{\left(CaCO3\right)}=0.5\;\left(mol\;eq^{-1}\right)\times \left[\Delta A_{T}+\Delta PO_{4}-\Delta NH_{4}+\Delta \left(NO_{3}+NO_{2}\right)\right]\times V_{SW}\times \rho_{SW}\times 100$$ (1)

where ΔA_T_ is the change in total alkalinity. Multiplication factor ‘0.5’ reflects the 2:1 relationship between the increase in A_T_ during CaCO_3_ dissolution. V_SW_ is the volume (L) of seawater in the incubation chamber and ρ_SW_ is seawater density (1.023 kg L^-1^). The multiplier ‘100’ incorporates the molecular mass of CaCO_3_.

A_T_ was measured using an Automated Spectrophotometric Alkalinity System (ASAS) [67] following the method outlined by Breland and Byrne (1993) [68] and Yao and Byrne (1998) [69]. This optical titration procedure has a remarkable high precision (± 0.7 μmol kg^-1^) making it possible to detect minor alkalinity fluctuations. A_T_ was measured immediately after sample collection. To compensate for a drift in A_T_ over the course of the experimental period, certified reference material (CRM; supplied by Dr. A. Dickson, Scripps Institution of Oceanography) was analyzed every 20 samples. Filtered (acrodisc: 0.2 μm pore size) nutrient samples were stored at -20°C until transport to the Royal Netherlands Institute for Sea Research (NIOZ) where they were analysed on a QuAAtro continuous flow analyzer (SEAL Analytical, GmbH, Norderstedt, Germany) following GO-SHIP protocol [70]. Final chemical erosion rates were expressed in mg CaCO_3_ cm^-2^ d^-1^ or convertible units and nutrient dynamics in μmol L^-1^ h^-1^.

### Determining sponge surface area and biomass

At the end of each experiment all cores were photographed to estimate the surface area covered by external sponge tissue. For α-stage (only single inhalant and exhalant papillae are visible on the surface) sponges, the area in between the outermost papillae was taken as a measure for infested surface area. For β-stage specimens (papillae are fused into an encrusting sheet) the total sponge covered surface on both the top and side of each core was used. Additionally, the cores used in the BW experiment were dissolved in acid (2 M HCl) after the final weighing to isolate the sponge tissue for wet and dry-weight (24 h at 60°C) measurements and to verify the absence of other macroborers (e.g. worms and molluscs).

### Statistics

All statistical testing was conducted in the R programming environment R 3.3.2 [71] using the packages “stats” and “gam”. Assumptions of normality and homogeny were checked visually and all collected data were fourth-root transformed to stabilize variances. Generalized additive modelling was implemented to predict the fluctuations in rates of chemical erosion as a function of time in ST incubations. Two-way ANOVA (species and diurnal cycle) testing complemented by post-hoc Tukey-HSD paired comparisons were used to examine variation in patterns of excavation among and within the six studied sponge species.

## Results

### Chemical erosion

**Fig 3:**
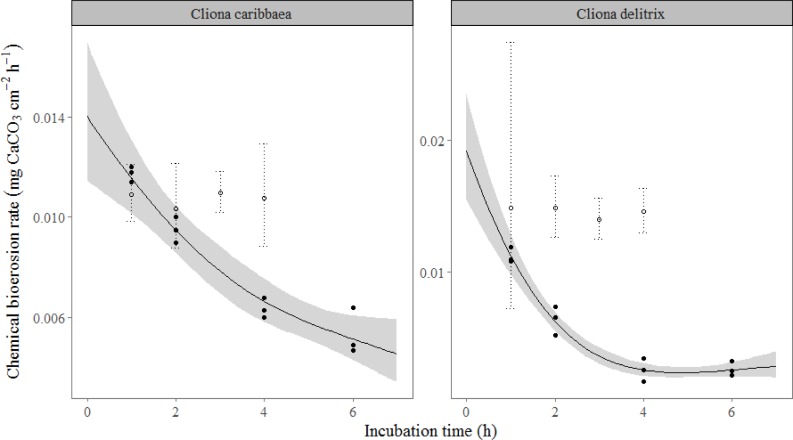
Modelled effect of incubation time on sponge chemical bioerosion rates. Black regression lines represent the modelled (GAM) mean dissolution in mg cm^-2^ h^-1^ by *Cliona caribbaea* (left panel) and *Cliona delitrix* (right panel) in standing incubations. 95% confidence limits are indicated by the grey band. Solid dots show the calculated mean dissolution rates at T_1_, T_2_, T_4_ and T_6_ (ST). Open dots represent rates derived from running flow-through incubations at T_1_, T_2_, T_3_ and T_4_, dotted lines here indicate 95% confidence limits. All rates are obtained using alkalinity anomaly techniques.

Comparison of chemical dissolution rates acquired through ST and FT incubation showed that duration of the ST incubation had a distinct effect on sponge chemical bioerosion. Dissolution rates by *C*. *caribbaea* (*F*_(3,8)_ = 33.9, *p* < 0.001) and *C*. *delitrix* (*F*_(3,8)_ = 27.3, *p* < 0.001) declined significantly as a function of the time cores were retained in the confined water body of the incubation chamber (Fig 3). After 2 hours (T_2_), rates had already decreased by 31% (*C*. *caribbaea*) and 70% (*C*. *delitrix*) compared to the modelled rate at T_0_, which is the hypothetical point at which the sponge should not yet experience any negative effect of being incubated. Comparison of dissolution rates between the ST and the FT during day-time (Fig 3) shows that the chemical dissolution extrapolated to T_0_ of the ST incubation, *C*. *caribbaea*: 0.014 mg cm^-2^ h^-1^ (95% CI: 0.011–0.017) and *C*. *delitrix*: 0.021 mg cm^-2^ h^-1^ (95% CI: 0.012–0.032), agrees with dissolution values obtained in FT incubation (Table 1, daily rates). A negative effect of incubation time was not observed for the two species in the flow-through methodology, at least not up to 4 h after the start of the incubation (Fig 3). This supports the long-term consistency of the FT methodology introduced here. Consequently it was decided to focus on measurements with the FT set-up.

**Table 1:** Summary of all calculated sponge bioerosion rates.

Species	Method	Chemical day (12 h)	Mechanical day (12 h)	Chemical night (12 h)	Mechanical night (12 h)	Total chemical (24 h)	Total mechanical (24 h)	Total erosion (daily)	Total erosion (annual)
*C*. *amplicavata*	Flow-through	0.08 (0.03–0.18)	1.54 (1.01–2.25)	0.12 (0.08–0.17)	1.26 (0.93–1.67)	0.20	2.80	3.00	10.95
* *	Buoyant weight							1.90 (0.93–3.49)	6.94 (3.40–12.74)
	BM (g DWsponge)							76.18 (16.44–230.2)	0.028
* *	7-day collection						2.52 (1.70–3.60) [0.77]		
*C*. *aprica*	Flow-through	0.04 (0.03–0.04)	0.85 (NA)	0.02 (0.02–0.02)	0.45 (0.33–0.60)	0.06	1.30	1.36	4.96
* *	Buoyant weight							0.71 (0.51–0.96)	2.59 (1.86–3.50)
	BM (g DWsponge)							66.40 (33.33–119.5)	0.024
* *	7-day collection						0.47 (0.37–0.60) [0.30]		
*C*. *caribbaea*	Flow-through	0.13 (0.12–0.15)	1.62 (1.32–1.96)	0.07 (0.06–0.07)	0.44 (0.74–1.50)	0.20	2.06	2.26	8.25
* *	Buoyant weight							0.29 (0.01–0.23)	1.06 (0.04–1.84)
	BM (g DWsponge)							21.99 (18.95–25.39)	0.008
* *	7-day collection						0.39 (0.30–0.52) [0.29]		
*C*. *delitrix*	Flow-through	0.18 (0.09–0.33)	2.08 (1.12–3.56)	0.30 (0.20–0.44)	0.87 (0.81–0.94)	0.48	2.95	3.43	12.52
* *	Buoyant weight							2.30 (1.09–4.31)	8.40 (3.98–15.73)
	BM (g DWsponge)							272.4 (95.98–622.5)	0.099
* *	7-day collection						1.79 (1.16–2.67) [0.50]		
*S*. *brevitubulatum*	Flow-through	0.11 (0.07–0.16)	0.69 (0.18–1.54)	0.16 (0.13–0.19)	1.02 (0.40–2.20)	0.27	1.71	1.98	7.23
* *	Buoyant weight							0.94 (0.39–1.94)	3.43 (1.42–7.08)
	BM (g DWsponge)							195.0 (53.75–516.1)	0.014
* *	7-day collection						0.69 (0.44–1.06) [0.26]		
*S*. *flavolivescens*	Flow-through	0.09 (0.06–0.12)	0.26 (0.14–0.44)	0.14 (0.09–0.21)	0.29 (0.18–0.45)	0.23	0.55	1.01	2.01
* *	Buoyant weight							0.17 (0.01–0.27)	0.62 (0.04–0.99)
	BM (g DWsponge)							7.00 (4.26–10.83)	0.003
* *	7-day collection						0.38 (0.29–0.50) [0.10]		

Bioerosion rates of six excavating sponge species acquired through different methodologies: flow-through incubation; buoyant weight; long-term (7 day) collection of mechanically removed material. The different elements (chemical and mechanical erosion both at day and night) that make up the total bioerosion are, when available, individually presented per method. Day and night rates are given for 12 h day and night. Erosion rates given in mg CaCO_3_ cm^-2^ d^-1^, with the exception of annual rates in the final column (given in kg CaCO_3_ m^-2^ y^-1^). Buoyant weight acquired rates converted to sponge dry weight (g) are given separately (BM) in mg (daily) and kg (annual) of removed CaCO_3_. Total mechanical erosion derived from the long-term collection experiment is given as the sum of sponge chips and non-chip CaCO_3_ material, the former is also provided individually in square brackets. 95% confidence intervals of the original rates are provided in round brackets. All rates have been corrected for the outcomes of the uncolonized control core experiments.

**Fig 4:**
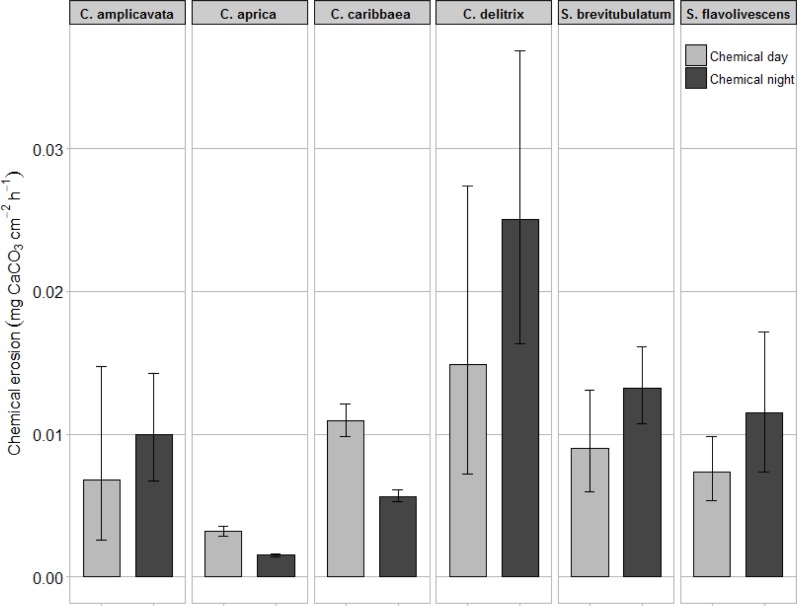
Chemical bioerosion. Hourly rates of chemical dissolution (mg CaCO_3_ cm^-2^ d^-1^) are given for the six studied sponge species (top). Both day (light grey) and night (dark grey) erosion rates are included. Rates are derived from alkalinity change in flow-through incubation. Error bars indicate the 95% confidence limits.

Chemical dissolution rates acquired through FT incubation revealed significant (*F*_(38)_ = 4.11, *p* < 0.01) variation among sponge species (Fig 4 and Table 1). Interspecific variation was predominantly associated with morphology resulting in significantly slower erosion rates for both gallery-forming species: *C*. *caribbaea* and *C*. *aprica*. These high density zooxanthellate sponges also showed clear diurnal disparity with significantly higher (*p* < 0.01 for both species) dissolution rates during the day (Fig 4). In *C*. *amplicavata*, *C*. *delitrix*, *S*. *brevitubulatum* and *S*. *flavolivescens* night erosion appeared to be higher than day erosion, although this was not significant for any of these species. Notably, the inflow water was slightly more basic during day-time (pH = 8.08 ± 0.001) compared to night-time (pH = 8.02 ± 0.007).

### Nutrient dynamics

Considerable nutrient accumulation (especially NH_4_^+^ and NO_3_^-^) occurred at day-time in ST (1 h) incubations holding *C*. *caribbaea* (Table 2). Accumulation of nutrients after 1 hour was largely prevented in the FT system (Table 2). Observed fluctuations in phosphate concentrations were largely negligible, there was essentially no indication for net uptake. In FT incubations a minor elevation in levels of ammonium and nitrate was observed during most incubations at night. During the day, these nutrients appear to be consumed in the majority of the incubations, including those containing non-infested cores. Nitrogen can be taken up by the sponge itself or by its zooxanthellae (i.e. *C*. *caribbea*, *C*. *aprica* and to a lesser extend *C*. *delitrix*). The observed decline in control incubations (containing uncolonized cores) indicates that there is likely also an effect on nutrient levels caused by micro-organisms in the incubation water. When considering the nutrient trajectories in the control water many of the observed dynamics in chambers containing sponges are largely dampened (Table 2).

**Table 2:** Summary of nutrient dynamics in the incubation chambers.

Species		n	NH_4_^+^	NO_2_^-^	NO_3_^-^	PO_4_^3+^
*C*. *caribbaea* (ST)	Day	3	2.06 ± 0.49	0.04 ± 0.03	0.62 ± 0.43	0.04 ± 0.03
	Night	2	0.87 ± 0.39	0.04 ± 0.01	0.2 ± 0.01	0.02 ± 0.004
*C*. *caribbaea* (FT)	Day	6	-2.41 ± 4.26	-0.04 ± 0.04	-1.92 ± 2.91	0.01 ± 0.01
* *	Night	2	0.98 ± 0.19	0.03 ± 0.00	0.42 ± 0.02	0.07 ± 0.01
*C*. *amplicavata* (FT)	Day	4	0.39 ± 0.14	0.02 ± 0.02	0.12 ± 0.05	0.01 ± 0.01
* *	Night	3	0.11 ± 0.21	-0.01 ± 0.01	0.21 ± 0.28	0.00 ± 0.00
*C*. *aprica* (FT)	Day	3	-0.15 ± 0.01	-0.02 ± 0.03	-0.04 ± 0.03	0.01 ± 0.00
* *	Night	2	0.36 ± NA	-0.003 ± NA	0.16 ± NA	0.03 ± 0.04
*C*. *delitrix* (FT)	Day	5	-0.21 ± 0.41	-0.03 ± 0.02	-0.37 ± 0.39	0.03 ± 0.05
* *	Night	3	0.12 ± 0.17	-0.03 ± 0.06	0.25 ± 0.07	-0.01 ± 0.02
*S*. *brevitubulatum* (FT)	Day	2	0.03 ± NA	-0.11 ± NA	-0.04 ± NA	0.01 ± 0.03
* *	Night	1	0.05 ± NA	-0.07 ± NA	0.14 ± NA	0.06 ± NA
*S*. *flavolivescens* (FT)	Day	3	-0.06 ± 0.25	-0.06 ± 0.03	0.78 ± NA	0.06 ± 0.02
* *	Night	2	-0.31 ± 0.13	0.03 ± 0.003	1.35 ± 0.04	0.06 ± 0.00
Uncolonized core (FT)	Day	1	-0.22 ± NA	-0.01 ± NA	-0.16 ± NA	-0.01 ± NA
	Night	1	0.03 ± NA	-0.04 ± NA	-0.03 ± NA	-0.01 ± NA

Change in nutrient (NH_4_^+^, NO_2_^-^, NO_3_^-^, PO_4_^3+^) concentration (μmol L sea water^-1^) after one hour of flow-through (FT) incubation. Concentrations are given for all six species and uncolonized control cores at day and night. Nutrient dynamics are also provided for *C*. *caribbaea* in 1 h standing incubation (ST). Rates are given with their standard deviation. n: number of nutrient samples. No standard deviation is given (NA) when only 1 nutrient value was available.

### Mechanical erosion

**Fig 5:**
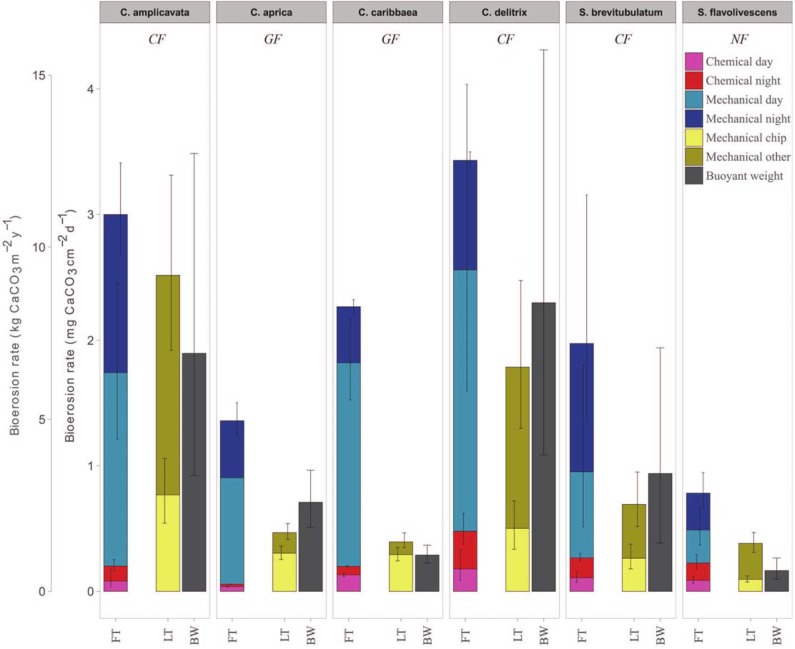
Total sponge bioerosion. Final rates of total erosion by the six sponge species (top) acquired through flow-through incubation (FT) and buoyant weight loss over a three week period (BW). The different bioerosive components (FT) are based on a 12 h day/night diurnal cycle. Mechanical bioerosion rates based on long-term collection are provided separately (LT). Here, a distinction is made between actual sponge chips and other CaCO_3_ fragments that have been dislodged through sponge bioeroding activity. Rates are given in mg CaCO_3_ cm^-2^ d^-1^ and kg CaCO_3_ m^-2^ y^-1^. Error bars indicate the 95% confidence intervals for each individual element. Species and boring strategies are provided at the top of each panel, *CF*: cavity-forming, *GF*: gallery-forming; *NF*: network-forming.

**Fig 6:**
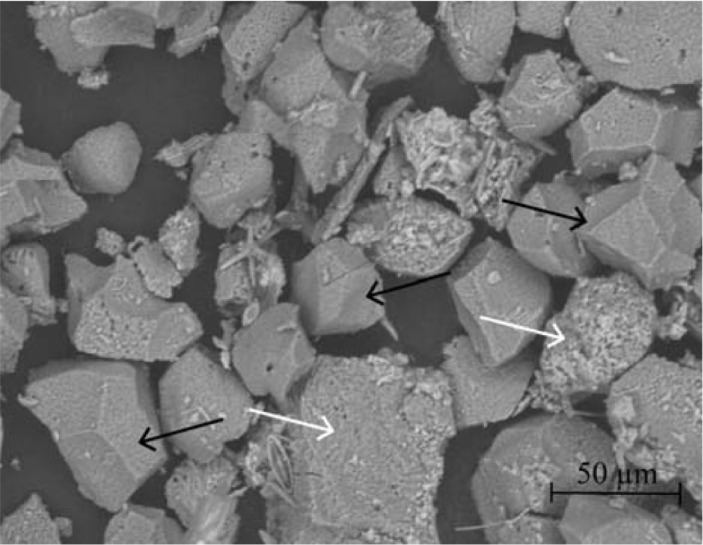
Scanning Electron Microscopy (SEM) image of material produced by *C*. *caribbaea*. The provided image shows only a fraction of the total material produced over the 7-day collection experiment. Black arrows: some characteristic sponge chips, white arrows: other CaCO_3_ fragments. Scale is provided in lower-right corner.

The mechanical fraction of sponge bioerosion was based on the weight of the particulate material collected in the incubation chamber after 1 h FT incubation and in the funnels for the long-term (7 day) collection experiment, yielding two estimates per species (Fig 5 and Table 1). Although both estimates are in a similar range, rates derived from FT incubation are consistently higher. SEM analyses of 7-day filters did revealed that, depending on the species, 17% to 45% of the collected material (long-term) could be attributed to actual sponge chips (Fig 6). The remaining material consisted mostly of non-chip CaCO_3_ fragments with some occurrences of foreign planktonic material or spicules. The fraction of non-chip material that was dislodged by sponge could not be traced back directly to boring activity and thus represents an estimate (i.e. total non-chip CaCO_3_ fragments minus the fragment production by non-infested cores). CaCO_3_ fragment removal differed significantly among species (*F*_(30)_ = 4.43, *p* = 0.003), with most material removed by *C*. *delitrix* and *C*. *amplicavata*. Similar to the chemical fraction, there appears to be a strong diurnal pattern in mechanical substrate removal in both symbiont-rich species as well as the symbiont-poor *C*. *delitrix* (Table 1). ANOVA testing of the FT results showed that mechanical erosion at night was significantly lower only for *C*. *caribbaea* (*F*_(1,5),_ = 59.6, *p =* 0.016). It should be noted, however, that only one sample was available for *C*. *aprica* at day which strongly hampered proper statistical testing. Mechanical erosion by the other four species did not differ significantly at day or night (*p* > 0.05).

### Total bioerosion

Total bioerosion (i.e. chemical and mechanical) quantified from FT incubations was consistently higher than BW acquired rates (Table 1 and Fig 5). For *C*. *amplicavata*, *S*. *brevitubulatum* and *S*. *flavolivescens* both rates are still within a similar range. However, the discrepancy between methods was particularly pronounced in both symbiont-rich species (*C*. *caribbaea* and *C*. *aprica*) and *S*. *flavolivescens*. Nevertheless, the observed patterns of inter-specific variation were highly comparable among both approaches, with highest erosion rates found for the three cavity-forming species. Lowest rates are consistently observed for network-forming *S*. *flavolivescens*. There appears to be considerable variation in BW rates among cores of the same species (suggested by the large confidence intervals), in particular among the cavity-formers (Fig 5). BW rates converted to mg CaCO_3_ g DWsponge^-1^ d^-1^ based on the dry weight of the sponge tissue in each core yielded considerable similarity in patterns of inter-specific variation to rates based on surface area (Table 1 and S1 Fig). The cavity-forming *C*. *amplicavata*, however, appear to be an exception to this apparent surface area–biomass relation. FT estimates indicate that for five out of the six species the mechanical fraction accounted for the vast majority of total bioerosion (86%–96%). Interestingly, for the network-forming *S*. *flavolivescens* chemical dissolution (46%) and mechanical fragment removal (54%) contributed almost equally.

## Discussion

We quantified the bioerosive capacity of six excavating sponge species commonly found on the reefs of Curaçao. This is the first time individual chemical and mechanical rates and total bioerosion rates are provided simultaneously for the majority of these species. Overall, *Cliona delitrix* and *Cliona amplicavata* (both cavity-forming species) were found to have the highest boring rates. *C*. *delitrix* is indeed known to be among the most destructive bioeroding sponges on many Caribbean reefs [72]. It should be pointed out that *C*. *amplicavata* was found solely in *Acropora* spp. rubble which is of higher skeletal density than *Orbicella* spp. [61]. Substratum density can strongly affect bioerosion, where denser substratum generally leads to higher erosion rates (e.g. [42, 43]). Erosion by *C*. *caribbaea* and *C*. *aprica*, both adopting a gallery-forming strategy, was found to be considerably lower compared to cavity-formers. Nonetheless, these species, together with *C*. *delitrix*, are the dominant excavating sponges on the shallow reefs of Curaçao and many sites in wider Caribbean region [39, 57, 58] and thus contribute significantly to total macrobioerosion. *S*. *flavolivescens*, previously undescribed to occur on Curaçaoan reefs, appears to be the weakest eroder of all sponge species studied here. This network-forming species adapts a different boring strategy (i.e. network-forming) from those outlined by Murphy et al. (2016) [27].

### Methodological considerations

Comparison of the chemical bioerosion in the flow-through (FT) and standing (ST) approach reveals that an incubation time of 1 h or longer in the ST incubation chambers was too long to estimate the purported “real” bioerosion rate. Bioerosion steeply dropped in time, suggesting that food depletion and or waste accumulation inhibited chemical bioerosion. Neumann (1966) [45] suggests that sponges can bore more actively in a high water flow environment, possibly due to a larger supply of fresh sea water. Being known as highly efficient filter-feeding organisms (e.g. [73]) it is not surprising that these sponge have the capacity to rapidly consume the available food sources, both particulate and dissolved [74] in any limited body of water. Consequential stress could significantly affect the metabolism of the sponge, possibly redirecting energy away from secondary processes such as boring and focussing more on vital functions like maintaining a high pumping velocity. Extrapolation of rates determined in the ST to T_0_ revealed chemical dissolution rates comparable to rates found in the FT incubators. This implies that whenever the ST set-up is preferable, the optimal incubation times should be determined in advance in the devices used and individually for each sponge species. To date, standing incubation techniques generally resulted in lower rates for chemical erosion, but have also predominantly been used to test the effect of environmental change on the boring efficiency through sea water manipulation ([40] and references therein). Although in most of these studies the authors looked at relative variation in rates as a response to treatment, the acquired rates should be interpreted with some degree of caution. The processes acting inside these enclosed chambers are largely unknown and unpredictable. More desirable, therefore, would be the use of FT incubation, since here the effect of incubation time on measured variables appears to be negligible (Fig 3 and Table 2).

CaCO_3_ fragment production (including sponge chips) in the FT incubation chambers was generally higher than the fragment production by hanging cores in a large aquarium. Webb et al. (2017) [34] propose that elevated levels of Ca^2+^ in incubation water (ST) following chemical dissolution of CaCO_3_ may cause contraction of the conductive pathway of the sponge. Such a contraction could result in an initial pulse ejection of the chips present within the sponge tissue, resulting in excessively high production over the relatively short incubation. However, in our FT incubations accumulation of Ca^2+^ will have been limited because the incubation water was constantly refreshed. Since the discrepancy between methods was most pronounced in both symbiont-rich species (*C*. *caribbaea* and *C*. *aprica*) and *S*. *flavolivescens*, it is more likely that the reversed orientation of the hanging cores may have reduced the boring capacity of these sponges following limited light availability. In addition, this methodology (hanging cores) might be more prone to loss of a fraction of the produced silt-sized fragments due to water movement in the holding tank [35]. Overall, there is still hardly any information available on the mechanisms underlying mechanical erosion or the expelling of dislodged material by excavating sponges. Both approaches (short and long term collection), however, can provide better insight in the mechanisms underlying mechanical erosion and the expelling of dislodged material by excavating sponges.

For most species, final FT rates are comparable to rates acquired gravimetrically based on the loss in buoyant weight (BW) of the invaded cores (Fig 5). Strongest variation between methodologies was found in *C*. *caribbaea* and *C*. *aprica* and *S*. *flavolivescens*. Similar to the long-term fragment collection, the relatively low BW estimates are likely the result of the reversed orientation of the cores (see previous paragraph). This might also explain why for some hanging species mechanical erosion exceeded total bioerosion. CaCO_3_ fragment production was only measured in the first week. Possibly the boring capacity of the hanging sponges had further decreased in the two following weeks. We therefore argue that BW estimates in this study may reflect a slight underestimation of sponge bioeroding capacity and FT obtained rates should be considered more reliable. Up to now, however, conflicting studies generally found up to five times higher rates obtained through buoyant weighing compared to standing incubation derived estimates [34, 35]. The observed discrepancy has caused scepticism regarding the use of BW technique. Our results, however, support the use of the buoyant weight methodology, as described in previous studies, as a reliable approach to estimate rates of bioerosion and suggest that the previous implementation of standing incubation may often have resulted in underestimated rates. Nevertheless, to examine the effect that changing environmental conditions might have on bioerosion it is essential to consider both fractions independently. Particularly in view of the effect factors such as elevated levels of CO_2_ in ocean water have on the chemical dissolution capacity of the various sponge species. Preferably this is done in an incubation set-up where chemical erosion can be determined individually as well. Consequently, the methods presented here, with some minor improvements where necessary (read BW, long-term fragment collection), can and should be used concurrently depending on the underlying scientific aim.

### Bioerosion by different species

FT incubation revealed that the chemical bioerosive capacity of *C*. *caribbaea* and *C*. *aprica* was notably higher during the day (Fig 4). This is likely related to the presence of photosynthetic symbionts in these *viridis*-complex species. The photosynthetic capacity of intracellular symbiotic zooxanthellae was pinpointed as a possible primary source of carbon for these sponge species [65, 75, 76]. A consequential diurnal variation in rates of bioerosion following light availability has previously been described for *C*. *caribbaea* in St. Eustatius (Lesser Antilles) [34], *Cliona varians* in the Florida Keys [65] and *Cliona orientalis* in Australia [64, 77], all of which are species in the *C*. *viridis* species complex. *Symbiodinium* spp. in *C*. *delitrix*, present in much lower densities [56], did not seem to cause significant variation in chemical rates between day and night. Accordingly, this species appears to rely predominantly on heterotrophic feeding to fuel bioerosion. Similarly, no significant diurnal pattern was found for any of the other species, although chemical dissolution by these species does appear to be somewhat lower during the day. Possibly, these species benefited from the small decrease in sea water pH at night. Indeed, photosynthesis during the day-time and respiration at night by primary producers is known to cause diurnal variation in pH [78]. The latter suggests that these sponges might respond more firmly than symbiont-rich species to a decreasing sea water pH as a result of ocean acidification. This corresponds to the findings of Webb et al. (2017) [34] that at night dissolution by *C*. *caribbaea* only started increasing at a strongly elevated pCO_2_ of the incubation water. The absence of a clear diurnal pattern in azooxanthellate species is in compliance with findings of Schönberg (2008) [79] who studied rates of *Cliona celata* in the North Sea, but also with Zundelevich et al. (2007) [36] who studied the zooxanthellate *Pione vastifica* in the Red Sea.

Rates obtained through FT incubation show that mechanical removal of invaded substratum contributed most significantly (86–95%) to total estimated bioerosion rates. Only for *S*. *flavolivescens* (network-forming) chemical and mechanical erosion was approximately equal. A detailed visual inspection of *S*. *flavolivescens* specimens, however, revealed a large quantity of CaCO_3_ particles to be present in its dense tissue network. It is possible that this species retains most of the mechanically removed fragments, causing an underestimation of mechanical erosion when using methods as presented here. With the exception of *S*. *flavolivescens* the ratio between dissolution and mechanical substrate removal resembles that described in the majority of the published literature, where it is suggested that mechanical dissolution contributes between 83% to 98% to total bioerosion by various studied excavating sponge species (e.g. [29, 32, 33–35]). Based on our findings there appears to be no obvious variation in this ratio among species adapting different boring strategies. However, we did observe some diurnal variation in the mech:chem ratio, with chemical erosion having a slightly more pronounced contribution to total erosion at night. This was especially clear for *C*. *delitrix* (chemical contribution: day: 8%, night 26%) and might be a result of decreased pH at night-time. This suggests that the mech:chem ration might shift with the predicted ocean acidification scenarios. The portion of mechanically removed fragments that could be attributed to the characteristic sponge chips was limited compared to the total collected CaCO_3_ for all six species. This was particularly evident in the three cavity-forming species (C. *amplicavata C*. *delitrix* and *S*. *brevitubulatum*) and *S*. *flavolivescens*, where chips accounted for a mere 17%–24%. CaCO_3_ material produced by *C*. *aprica* (74%) and *C*. *caribbaea* (64%) contained considerably more sponge chips, suggesting chip production may be linked to the adopted excavating strategy. Although chips are with great certainty removed by the sponge itself, many of the remaining CaCO_3_ fragments are most likely also the dislodged as a result of sponge boring activity since substantially less of this material was present on filters of uncolonized cores.

The sum of chemical and mechanical rates (both FT) realized final estimates of total annual bioerosion for all six studies species: 10.95 kg m^-2^ y^-1^ (*C*. *amplicavata*); 4.96 kg m^-2^ y^-1^ (*C*. *aprica*); 8.25 kg m^-2^ y^-1^ (*C*. *caribbaea*); 12.52 kg m^-2^ y^-1^ (*C*. *delitrix*); 7.23 kg m^-2^ y^-1^ (*S*. *brevitubulatum*) and 2.01 kg m^-2^ y^-1^ (*S*. *flavolivescens*). With the exception of *C*. *amplicavata* all rates presented here is based on bioerosion of *Orbicella* spp. coral substratum which is of ‘average’ density and the most ubiquitous substratum on many reefs throughout the Caribbean. The considerable variation in published bioerosion rates ([40]: S3 Table) can largely be attributed to factors such as the density of the substratum (e.g. [41, 42]), but also studied species, developmental stage and applied methodology. Acker and Risk (1985) [33] provided total erosion rates for *C*. *caribbaea* in *Orbicella* spp. (8.0 kg m^-2^ y^-1^) that are comparable to rates presented here. More recently, a considerably lower rate (1.7 kg m^-2^ y^-1^) for total erosion by *C*. *caribbaea* was described for *C*. *caribbaea*, but in less dense *Diploria* spp. substratum and based on 6 h standing incubation [34]. Similar rates (2.2–2.5 kg m^-2^ y^-1^) were described for its ‘Pacific equivalent’ *C*. *orientalis* in massive *Porites* spp. coral [35, 52]. Both Fang et al. (2013b) [35] and Webb et al. (2017) [34], however, found 3 to 5 times higher rates using the buoyant weight technique which results in rates that are similar to our findings. Estimates described for *C*. *aprica* range higher (7.0 kg m^-2^ y^-1^) than our findings but it should be noted that this is based on erosion of extremely dense Conch shells [44]. Substantially higher bioerosion rates were found for other *C*. *viridis*-complex species in substratum that is substantially denser than that of *Orbicella* spp. skeleton: *C*. *orientalis* (up to 17.6 kg m^-2^ y^-1^) [42] and *C*. *varians* (up to 22.8 kg m^-2^ y^-1^) [65]. Of the sponges studied here, *C*. *delitrix* is generally considered among the most aggressive eroding species [72, 80] and our data appears to support this claim. The observed eroding capacity of *C*. *delitrix* in the α-stage (this study) infers the bioeroding potential of *C*. *delitrix* since the β-stage is deemed considerably more destructive [81]. Next to *C*. *delitrix*, substantially high rates were only found for *C*. *amplicavata*, but in *Acropora* spp. rubble which has a considerably denser skeletal structure than *Orbicella* spp. Murphy et al. (2016) [27] are the only study to have assessed the bioerosive capacity of *S*. *brevitubulatum*, adapting a completely different approach including substrate density and lateral expansion rate. Following their analogy a hypothetical sponge would bore with a rate of 4.4 kg m^-2^ y^-1^ in carbonate substrate with a density of 1.7 g cm^-3^. This is in range with our estimates in substratum of comparable density. It is noteworthy that the estimated rates for *S*. *brevitubulatum* as presented in this work may not fully reflect the boring capacity of this species considering that most of the soft internal tissue needed to be removed to prevent tissue decomposition. To our knowledge, the bioeroding capacity of *C*. *amplicavata* has not previously been determined. Also, no representative data is available for *S*. *flavolivescens* or any species that adapts a similar excavating strategy.

Overall, patterns of inter-specific variation were strikingly similar when related to either surface area or biomass of the sponge. This would suggest a consistent link between surface area and internal biomass of the sponge with regards to bioerosive activity, which is in contrast to the few existing preceding studies addressing this issue [40, 44, 82]. Nonetheless, it remains difficult to properly relate surface area to internal biomass of the sponge and we only quantified sponge biomass in three cores per species. Elaborate research on this specific relationship is crucial to properly estimate the contribution of excavating sponges to the reef carbonate budget.

We provide a comprehensive comparison of methods to quantify chemical and mechanical erosion and tentatively endorse methodologies that could be implemented widely in an attempt to support standardized data collection. The ‘novel’ FT technique described here is assumed to more adequately mimic natural *in situ* conditions, thus providing a more reliable representation of the bioerosive capacity of the excavating sponge community. We therefore encourage the use of flow-through incubation techniques next to buoyant weighing techniques, in particular when the aim is to determine the chemical fraction of bioerosion separately. If for some reason standing incubation techniques are preferred, the length of the incubation should be minimized or at least two time points should be included so that the signal at T_0_ can be predicted. It should be noted that determining mechanical erosion based on collected particulate material will always be prone to underestimation because loss of particles in the smallest size class can not be fully excluded. This is the first study to estimate rates for such an extensive range of species encompassing the relevant majority of the sponge bioeroding community of Curaçao and the wider Caribbean. This includes multiple α-stage species which, to date, have largely been ignored because their distinct papillate-morphology complicates linking assessed rates to the infested surface area [40]. Although we recognize that these data cover only part of the possible variation in rates of bioerosion caused by inter-specific and intra-specific variation, developmental stage, successional stage, density of the invaded substrate, reef-zone [43] and demography it can be considered an important addition to the currently still limited knowledge on the bioerosive impact of the excavating sponge community on coral reefs. The data presented here, combined with previously published and future work can also be included in standardized surveys, such as the *ReefBudget* methodology [39], resulting in a more complete and correct image of the carbonate budget of Caribbean coral reefs.

## Supporting information

S1 Fig Daily removed CaCO_3_ (mg) per gram of dry weight sponge tissue. Provided rates are based on the loss in buoyant weight of sponge infested cores (n = 3 per species) over a three week period. Sponge tissue was dried for 24 h at 60°C. Rates are given for all six studied species: *Cliona amplicavata* (cavity-forming), *Cliona aprica* (gallery-forming), *Cliona caribbaea* (gallery-forming), *Cliona delitrix* (cavity-forming), *Siphonodictyon brevitubulatum* (cavity-forming) and *Suberea flavolivescens* (network-forming). All rates include a correction for the outcomes of uncolonized control core experiments. Error bars represent the 95% confidence limits.
(EPS)

S1 Table Overview of the data supporting the results presented in this article. (XLSX)
